# Evaluation of an operational malaria outbreak identification and response system in Mpumalanga Province, South Africa

**DOI:** 10.1186/1475-2875-7-69

**Published:** 2008-04-27

**Authors:** Marlize Coleman, Michael Coleman, Aaron M Mabuza, Gerdalize Kok, Maureen Coetzee, David N Durrheim

**Affiliations:** 1 School of Animal, Plant & Environmental Sciences, University of the Witwatersrand, Johannesburg, Gauteng, South Africa; 2 Liverpool School of Tropical Medicine, Pembroke Place, Liverpool, L3 5QA, UK; 3 Medical Research Council, Durban, South Africa; 4 Mpumalanga Department of Health, 66 Anderson Street, Nelspruit, 1200, South Africa; 5 Vector Control Reference Unit, National Institute for Communicable Diseases, National Health Laboratory Service, 1 Modderfontein Road, Sandringham, 2131 Johannesburg, South Africa; 6 SA Research Chair in Medical Entomology & Vector Control, School of Pathology, University of the Witwatersrand, Johannesburg, South Africa; 7 Hunter New England Population Health and Hunter Medical Research Institute, Locked Bag 10, Wallsend, 2287, Australia

## Abstract

**Background and objective:**

To evaluate the performance of a novel malaria outbreak identification system in the epidemic prone rural area of Mpumalanga Province, South Africa, for timely identification of malaria outbreaks and guiding integrated public health responses.

**Methods:**

Using five years of historical notification data, two binomial thresholds were determined for each primary health care facility in the highest malaria risk area of Mpumalanga province. Whenever the thresholds were exceeded at health facility level (tier 1), primary health care staff notified the malaria control programme, which then confirmed adequate stocks of malaria treatment to manage potential increased cases. The cases were followed up at household level to verify the likely source of infection. The binomial thresholds were reviewed at village/town level (tier 2) to determine whether additional response measures were required. In addition, an automated electronic outbreak identification system at town/village level (tier 2) was integrated into the case notification database (tier 3) to ensure that unexpected increases in case notification were not missed.

The performance of these binomial outbreak thresholds was evaluated against other currently recommended thresholds using retrospective data. The acceptability of the system at primary health care level was evaluated through structured interviews with health facility staff.

**Results:**

Eighty four percent of health facilities reported outbreaks within 24 hours (n = 95), 92% (n = 104) within 48 hours and 100% (n = 113) within 72 hours. Appropriate response to all malaria outbreaks (n = 113, tier 1, n = 46, tier 2) were achieved within 24 hours. The system was positively viewed by all health facility staff. When compared to other epidemiological systems for a specified 12 month outbreak season (June 2003 to July 2004) the binomial exact thresholds produced one false weekly outbreak, the C-sum 12 weekly outbreaks and the mean + 2 SD nine false weekly outbreaks. Exceeding the binomial level 1 threshold triggered an alert four weeks prior to an outbreak, but exceeding the binomial level 2 threshold identified an outbreak as it occurred.

**Conclusion:**

The malaria outbreak surveillance system using binomial thresholds achieved its primary goal of identifying outbreaks early facilitating appropriate local public health responses aimed at averting a possible large-scale epidemic in a low, and unstable, malaria transmission setting.

## Background

Malaria is the most important parasitic and vector borne disease globally accounting for 300 million episodes and between one and three million deaths a year [1,2]. It is estimated that 100 million people are at risk from malaria epidemics [3] and the potential value of predicting malaria outbreaks and epidemics has been recognized [4]. Epidemics generally refer to increases in disease in relatively large populations, while outbreaks are considered to be more focal in occurrence and often precede an epidemic by a number of weeks [5]. The implementation of methods for early detection of an increase in malaria cases is advocated by the World Health Organizaiton (WHO) [6].

Effective outbreak containment demands prompt recognition and reporting of an unexpected increase in malaria cases to those responsible for control activities [7]. This is a challenge in under-resourced malaria-affected African regions where limited public health information systems exist [8]. It is critical in these areas that tools to assist programmes and responses are successfully deployed at operational level [9].

Stable endemic malaria characterizes much of Africa, however the fringes of the malaria affected area and highland areas (e.g. in Kenya and Uganda) are prone to epidemics. Climatic variation, including El Niño events, have been associated with the occurrence of malaria epidemics in these fringe areas [10,11]. Thus, the WHO malaria early warning system, which is climate based, is useful at regional level for alerting countries to a possible increased risk of malaria epidemics, although this has not been universally successful [6,8].

Malaria endemicity is not homogenous at country level. Complementary local systems are required to allow rapid redistribution of local resources to areas experiencing outbreaks. A number of complex models have been proposed to provide a local level warning based on climate, remote sensing models, syndromic surveillance and incidence patterns, [9,12-15] but their complexity suggests that successful and sustained programme level adoption will be challenging.

Malaria remains a public health problem in the north-eastern part of South Africa, including the low altitude areas of Limpopo Province, Mpumalanga and north-eastern KwaZulu-Natal [16,17]. Malaria risk is relatively low compared to other hyper- and holoendemic areas of sub-Saharan Africa and thus immunity does not develop in the population at risk. Mpumalanga Province, which borders Mozambique and Swaziland, is a predominantly rural area with a population of approximately four million. 95% of malaria cases are due to *Plasmodium falciparum *infection. This area has historically experienced malaria outbreaks and epidemics with attendant relatively high mortality [18]. Vector control in Mpumalanga is mainly by indoor residual spraying (IRS) with DDT on traditional mud, unplastered and water based painted surfaces and a synthetic pyrethroid for enamel painted wall surfaces. The introduction of definitive diagnosis using rapid diagnostic tests and mandatory reporting of cases [19,20] made the development of a malaria incidence based outbreak identification system feasible. Successful local implementation of a health facility-based syndromic outbreak detection and response system [21] suggested that a simple clinic based malaria outbreak detection and reporting system might be feasible if clinic staff were adequately trained, supported and appreciated the value of their contribution.

This paper describes the implementation of a three-tier malaria outbreak identification system using historical binomial thresholds to prompt early outbreak response and direct focal malaria control interventions. Retrospective data were used to compare the performance of the binomial threshold method to other currently recommended statistical approaches.

## Methodology

### Study region

**Figure 1:**
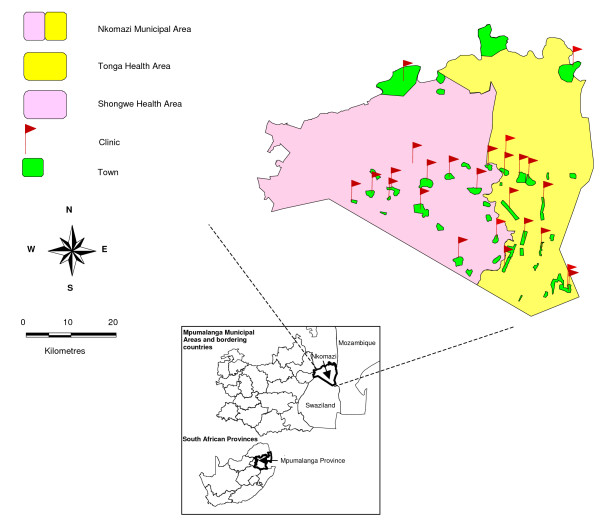
Nkomazi study area, Mpumalanga Province, South Africa.

Twenty five health facilities and 42 town/villages in the highest risk Nkomazi municipal area in Mpumalanga province (Figure 1) fully implemented the outbreak identification system in July 2005. Malaria is seasonal in this area with the high-risk period occurring from September to April, during the period of high humidity and rainfall. Estimated population at risk in this area is 380,000 (Statistics South Africa 2005).

### Threshold calculation

The binomial exact calculation [22] was used to determine individual thresholds for each health facility and town/villages by week. Exact confidence intervals (95% and 99%) were used to calculate level 1 and level 2 outbreak thresholds respectively.

Town/village populations [23] and catchments populations for each health facility (Mpumalanga Department of Health, unpublished data) were used to determine the denominator populations at risk of malaria. Expected cases were derived from malaria notifications by facility and source of infection at town/village level by week for the previous five malaria seasons (July to June) using weightings derived during a nominal group exercise. During a three round e-mail Delphi survey, eleven malaria experts from the South African Medical Research Council, World Health Organization, South African National Department of Health and Mpumalanga Provincial Department of Health provided their weighting and rationale for the differential proportional contribution of previous seasons in predicting the next season's total malaria notifications.

### Description of the outbreak identification and response system

#### Tier 1

**Figure 2:**
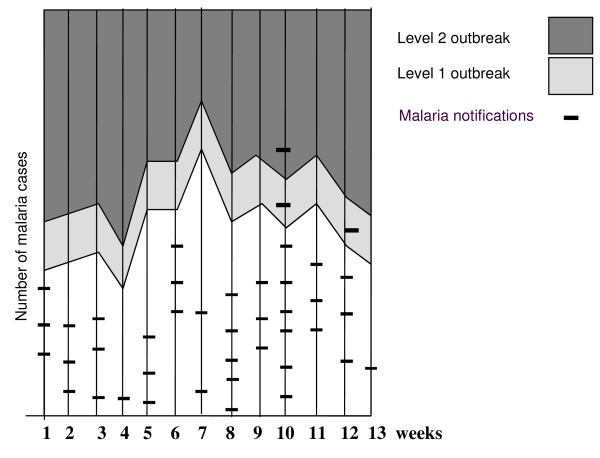
An example of a clinic outbreak identification chart.

Charts with weekly bi-level outbreak thresholds (Figure 2) were developed for each health facility allowing daily tallies of confirmed cases to be cumulatively charted against the weekly threshold. The mandatory paper notification forms were also completed for each case. When outbreak level 1 or 2 thresholds were exceeded the malaria control programme (MCP) was notified by clinic staff and stock levels of malaria treatments and rapid diagnostic tests (RDTs) verified to ensure that adequate supplies were available for outbreak case management.

#### Tier 2

MCP field staff received tables (Table 1) with the weekly thresholds pre-entered for each town/village within their area of responsibility. Tallies were based on the most likely source of infection town/village as completed on each notification form from health facilities. Notified weekly town/village cases were compared to weekly thresholds. Where thresholds were exceeded, field staff conducted home visits to confirm the likely source of infection and performed screening amongst individuals in the case household and neighbouring households. If a person was symptomatic, a RDT was performed and all individuals with positive RDTs were referred to the nearest health facility. Blood slides were taken from remaining household members and screened at the provincial malaria laboratory. All positive individuals were contacted and referred to health facilities for treatment within 48 hours.

**Table 1:** An example of a town/village threshold table

**Kamhlushwa Town**					
		**Thresholds**			

**Week No.**	**Date**	**Level 1**	**Level 2**	**Cases**	**Total weekly cases**

27	3/7/2006–9/7/2006	3	5	/	1
28	10/7/2006 – 16/7/2006	2	4		0
29	17/7/2006 – 23/7/2006	3	5	//	2
30	24/7/2006 – 30/7/2006	3	5		0

If level 1 or 2 outbreak thresholds were exceeded at town/village level, environmental assessment was conducted to identify local mosquito breeding sites [24]. Larviciding was performed as required and local coverage with indoor residual spraying (IRS) confirmed. If a level 2 outbreak threshold was exceeded for more than one successive week, additional IRS was considered in the town/village.

#### Tier 3

All malaria case information was entered into the Provincial Malaria Information System within a week of notification and outbreak detection algorithms by source location (town/village) were automatically run as each case was entered. If either threshold was exceeded an email alert was automatically sent to the relevant MCP staff members and their managers. This allowed performance monitoring of tier 2 responses.

### Evaluation

The binomial exact thresholds used in this identification system were retrospectively compared to the WHO recommended threshold mean +2 standard deviation [14,25] and the Centres for Disease Control and Prevention recommended cumulative sum [13] during the 2003/2004 season when a local malaria outbreak was experienced.

Uptake and usefulness of the system was evaluated by administering a structured questionnaire after each implementation season (2004/2005 and 2005/2006) at all primary health care facilities. A season refers to the period July to June.

## Results

In July 2004, the outbreak identification and response system was implemented in 12 primary health care facilities, 20 town/villages and then expanded in July 2005 to include 13 more health facilities and 22 town/villages in the Nkomazi municipal area (Figure 1).

During the 2004/2005 season a total of six outbreaks were reported from three (25%) health facilities. Only one (8.3%) level 2 outbreak was reported. All outbreaks were reported within 48 hours and occurred between August 2004 and January 2005 (Table 2). Response by the malaria field staff occurred within 24 hours of reporting. No stock-outs of drugs or RDTs were experienced.

**Table 2:** Malaria outbreaks by month, 2004/2005 season

**2004/2005**				
	**Level 1**		**Level 2**	

**Month**	**Health Facility**	**Town/Village**	**Health Facility**	**Town/Village**

**July**	0	0	0	0
**August**	4	1	1	0
**September**	0	1	0	0
**October**	0	2	0	1
**November**	0	0	0	0
**December**	0	0	0	0
**January**	1	1	0	0
**February**	0	0	0	0
**March**	0	0	0	0
**April**	0	0	0	0
**May**	0	0	0	0
**June**	0	0	0	0
**Total**	**5**	**5**	**1**	**1**

In the same season five (22%) town/villages reported level 1 outbreaks, and one a level 2 outbreak, all between August 2004 and January 2005 (Table 2). Only one town/village experienced a level 1 outbreak at both health facility and town/village level simultaneously. All cases were followed up and epidemiological field investigations conducted by field staff identified four additional patients by testing neighbours of index cases. Fifteen breeding sites were identified and larviciding performed with an organophosphate.

During the 2005/2006 season, 107 outbreaks were identified from health facilities. Twenty (80%) and 11 (44%) health facilities reported level 1 and level 2 outbreaks respectively. This differed significantly when compared to the first season (P = 0.0005; P = 0.0163 respectively). All outbreaks were reported within 72 hours and the majority (n = 91, 85%) within 24 hours. They occurred throughout the season (Table 3). Of the 77 level 1 outbreaks identified 30 (39%) became level 2 outbreaks. Review of stock inventories and routine ordering indicated that health facilities would have exhausted malaria treatment and RDT stocks if there had been no warning and additional orders placed.

**Table 3:** Malaria outbreaks by month, 2005/2006 season

**2005/2006**				
	**Level 1**		**Level 2**	

**Month**	**Health Facility**	**Town/Village**	**Health Facility**	**Town/Village**

**July**	3	0	1	0
**August**	1	1	0	0
**September**	1	2	0	0
**October**	3	3	1	1
**November**	2	1	0	0
**December**	9	4	5	0
**January**	22	5	14	3
**February**	13	4	5	2
**March**	13	5	3	3
**April**	4	3	0	1
**May**	4	2	1	0
**June**	2	0	0	0
**Total**	**77**	**30**	**30**	**10**

In the same season 40 outbreaks were identified at town level between the months of August 2005 and May 2006 (Table 3). Nineteen (45%) town/villages reported outbreaks at level 1 and eight (19%) reported level 2 outbreaks. Focal larviciding was carried out in 27 town/villages where breeding sites were identified. Testing of neighbours of index cases identified 19 additional malaria cases. New cases were notified and referred to a primary health care facility for treatment.

The rapid response at tier 2 was verified by finding that 100% of email outbreak alerts (tier 3) were received after a response to an identified outbreak had already been initiated. Outbreaks and response activities were verified by field managers and malaria information managers during weekly operational meetings and by provincial management at monthly MCP meetings.

The use and acceptance of the charts at health facilities was assessed using a structured questionnaire during site visits to all participating health facilities. Charts were available for viewing at all health facilities and all respondents indicated that the chart was useful, with the majority satisfied with the design (n = 32, 86%). A total of 25 (68%) respondents claimed that the charts gave an awareness of malaria risk and 6 (16%) used the chart for regular stock orders of malaria treatment and RDT's (Table 4).

**Table 4:** Summary of dichotomous responses to outbreak identification and response charts by health facility for the 2004/2005 and 2005/2006 season.

**Question**	**Yes n (%)**	**No n (%)**
**Chart available?**	37 (100)	0 (0)
**Chart complete?**	31 (84)	6 (16)
**Chart could be made more user friendly?**	5 (14)	32 (86)
**Chart useful?**	37 (100)	0 (0)
**Prefer to use formulas to determine own thresholds?**	1 (3)	36 (97)

### Comparison of thresholds

The outbreak identification thresholds were compared using retrospective case data from the 2003/2004 outbreak in Dindela town/village, Nkomazi municipal area. Comparisons were made between the WHO recommended mean +2 standard deviation [14,25], the CDC recommended cumulative sum thresholds [13] and the binomial thresholds method described above.

This particular period was selected as the seasonal malaria incidence rate for Dindela town was 25%, being the worst outbreak season recorded for a town in Mpumalanga Province (Mpumalanga Department of Health, unpublished data). No outbreak identification system was in existence at that time.

The number of outbreak identifications for the specific season varied according to threshold tested: C-sum (n = 32), mean +2 standard deviation (n = 29), binomial outbreak level 1 (n = 18) and level 2 (n = 12). The number of false outbreaks (this being outside the 20 week outbreak period) detected by the different thresholds also varied. The binomial exact thresholds detected 1, C-sum 12 and the mean + 2 SD points 9. All except the binomial level 2 threshold identified the actual outbreak four weeks before occurrence. The level 2 outbreak thresholds only detected the actual outbreak when it commenced.

## Discussion

The binomial outbreak identification and response system provided rapid alerts to focal malaria increases that prompted targeted public health action in the high-risk malaria area of Mpumalanga province during the two seasons following introduction. The need for ensuring adequate diagnosis and prompt treatment is essential as the local population has no acquired immunity following five decades of malaria control that has limited malaria transmission to the summer months [26,27].

In southern Africa a number of countries have acknowledged their inability to implement the WHO recommended malaria early warning system [6] despite its stated aim that it provides a simple and practical outbreak alert system. The primary goal of an outbreak surveillance system is to ensure timely recognition of abnormal levels of disease [28]. However, surveillance must be followed with a timely response or it becomes an academic exercise of limited value [29]. The Mpumalanga malaria control programme, in recognising the need for a malaria outbreak alert system, developed the tools described in this paper for supporting programme operations.

A climatic early warning system [8,30,31] with long lead times add limited value to Mpumalanga malaria control as extensive IRS is implemented annually. Simple models have been demonstrated to perform well in other areas [32]. Reliable focal outbreak detection is possible in Mpumalanga because timely and good quality malaria notification data is available [33].

In many malaria epidemic fringe areas the three tier binomial alert system could provide valuable intelligence to guide rapid public health action that could limit the extent and duration of outbreaks. It could ensure that there are adequate diagnostic and treatment supplies, provide ongoing monitoring of the performance of programme staff and guide focussed control activities.

It was encouraging to find that primary health care facilities embraced the system and recognized the additional benefit of utilising the charts for routine stock orders of malaria treatment and RDT's. This may ensure its sustainability.

Epidemiological methods including the WHO mean + 2 SD [34] and CDC's cumulative sum [13] have previously been used for outbreak identification and response. Historical comparisons using data from during a known epidemic period indicated limited concordance between the binomial thresholds and the C-sum [13] and mean + 2SD thresholds [14,25]. The large number of false weekly outbreaks identified by the C-sum and mean + 2 SD thresholds during this retrospective analysis would likely make them impractical in the Mpumalanga situation as unnecessary and costly responses would be required by the MCP staff. Our findings compare to similar findings from an evaluation project in Kenya that questioned the operational validity of WHO thresholds [13].

The computerized system, implemented as the third tier automatically calculated outbreak thresholds at the beginning of a malaria season. It also provided a timely quality monitoring mechanism for supervisors and managers to verify field operations.

The outbreak system described here was developed for a low malaria transmission setting and has direct application in other regions with a similar transmission pattern. These are expected to increase as a larger number of countries invest in enhanced malaria control and elimination. Current initiatives to control malaria with support from the Global Fund and President's Malaria Initiative are delivering success stories in malaria control including the Lubombo Spatial Development Initiative [35] and Bioko programme in Equatorial Guinea [36,37]. These will result in expansion of the zones where malaria endemicity is unstable with increased need to identify and rapidly contain focal outbreaks. The binomial outbreak identification and response system piloted in Mpumalanga should be tested in these areas as it appears to provide early identification of malaria outbreaks that facilitate timely response to ensure that control gains are sustained.

## Authors' contributions

Marlize C carried out all the investigative work and analysis under the guidance of Maureen C and DND. Field work would not have been feasible without the assistance of AMM and GK. Michael C drafted the manuscript and corrected subsequent drafts to which all authors contributed.
